# Fluctuations in Tat copy number when it counts the most: a possible mechanism to battle the HIV latency

**DOI:** 10.1186/1742-4682-10-16

**Published:** 2013-03-05

**Authors:** Zoran Konkoli, Aldo Jesorka

**Affiliations:** 1 Department of Microtechnology and Nanoscience - MC2, Chalmers University of Technology, Gothenburg, Sweden; 2 Department Chemical and Biological Engineering, Chalmers University of Technology, Gothenburg, Sweden

## Abstract

The HIV-1 virus can enter a dormant state and become inactive, which reduces accessibility by antiviral drugs. We approach this latency problem from an unconventional point of view, with the focus on understanding how intrinsic chemical noise (copy number fluctuations of the Tat protein) can be used to assist the activation process of the latent virus. Several phase diagrams have been constructed in order to visualize in which regions of the parameter space noise can drive the activation process. Essential to the study is the use of a hyperbolic coordinate system, which greatly facilitates quantification of how the various reaction rate combinations shape the noise behavior of the Tat protein feedback system. We have designed a mathematical manual of how to approach the problem of activation quantitatively, and introduce the notion of an “operating point” of the virus. For both noise-free and noise-based strategies we show how operating point off-sets induce changes in the number of Tat molecules. The major result of the analysis is that for every noise-free strategy there is a noise-based strategy that requires lower dosage, but achieves the same anti-latency effect. It appears that the noise-based activation is advantageous for every operating point.

## Introduction

### HIV-1 latency is a serious problem that prevents eradication of the virus

Human immunodeficiency virus 1 (HIV-1), initially reported nearly 30 years ago, represents a major global health problem with millions of people infected [1,2]. One of the biggest problems with HIV-1 is that the virus can enter a dormant state and effectively “hide” from drug cocktails that are therapeutically administered. Several reviews have been written on the subject [3-13]. Latent viral reservoirs can persist for many years. Once a therapy is interrupted, the latent reservoirs remain as the source of eventually renewed infection. This behaviour has been identified as the key problem in eradicating HIV-1.

There are many reservoirs, e.g., cell types, in the body that can harbor the latent virus. CD4+ T-cells have been identified as one of the largest pools of the viral DNA. This is also one of the best characterized reservoirs [5]. Once a T-cell is infected, it can either become activated and produce new virus particles that will infect other cells, or it can enter an inactive state. In the inactive (latent) state the transcription of the viral DNA is silenced, despite the fact that the viral RNA has been reversely transcribed and inserted into the host DNA.

The process of entry into the latent state is rather complex since it is controlled by a sizeable number of processes which need to occur nearly at the same time [8-10,13]. There is a relatively low percentage of latently infected T-cells, being roughly one per 10^6^, though the frequency can be lower [13]. This also suggests that once the latent state is established it remains very stable, and spontaneous activation events are very rare. In order to activate a latent cell, the inactive cellular processes need to be reactivated, which is not likely to occur simultaneously. A large number of studies have been performed in the past with the goal to find a way of reliably activating the latent virus, as reviewed in [3-13].

The mechanisms responsible for the maintenance of the HIV-1 latency work mostly at the molecular level. Processes such as chromatin control, a shortage of host transcription factors that initiate transcription, the presence of molecules that slow down (or even block) polymerase elongation, transcriptional interference, DNA methylation, and insufficient transport of viral mRNA from the nucleus to the cytoplasm are some prominent examples. All known mechanisms have been reviewed in detail, for example in [8-10,13].

A small number of studies focused on the role of chemical (intrinsic) noise in establishing the latent state [14-17]. A generic conclusion extracted from these studies is that noise is detrimental for the latency decision. Noise comes from fluctuations in copy numbers of the proteins that are involved in the latency control. The level of noise in the system is partially modulated by host factors (proteins that are present in healthy cells) and partially with virus specific factors (proteins encoded by viral genetic machinery). Noise makes the latency decision stochastic (unpredictable) and the literature suggests that the noise driven inactivation occurs spontaneously.

In the present study the focus is placed on understanding how noise can be manipulated to reverse the latency decision, *i.e.*, to drive the activation process. This idea is fully in line with several previous findings and suggestions in the literature. For example, it has been clearly appreciated that noise plays a role in various cellular decision making processes [18,19], but the idea to use noise to steer cell fate decisions has never been seriously explored. It has been argued, merely on a general basis, that one should strive to control noise better in order to steer cell fate decisions [19]. In the context of the HIV activation it has been found that altering noise of the HIV genetic machinery can bias virus decision making towards productive replication or latency [20].

In our study the focus is on finding ways to manipulate noise in order to specifically facilitate the productive activation, which could become the foundation of medical treatment strategies in the future. To our knowledge, this way of approaching the eradication of the latent virus has not yet been much discussed in the literature.

The first goal of this study is to identify regions in parameter space where noise greatly influences the dynamics. We investigate fluctuations of copy numbers of several key proteins that are involved in the maintenance of the latent state of infected cells. Based on these insights, the second goal is to identify suitable medical strategies that can be used to harvest noise in order to achieve more efficient activation. These intuitive considerations will be addressed in a quantitative way by means of a rigorous mathematics.

### The Tat feedback loop is a main source of noise driving the activation of the latent virus

The Tat feedback loop has been identified as an important part of the HIV gene expression machinery, reviewed in [3-5,7] and others. The main biological function of the Tat loop is to accelerate viral RNA production which further increases the number of viral particles in the infected cell. The presence of Tat molecules in the cell increases the transcription rate by roughly two orders of magnitude. This normally leads to cell lysis and continued infection of other cells. A lack of Tat molecules in latently infected cells has been identified as a strong barrier to activation of the latent cell [8-10,13].

Interestingly, the loop appears to be important for noise driven entry into the latent state [14-17]. If viral DNA integrates into regions of high basal transcription then noise does not play such a big role. However, if it integrates in regions of low basal transcription, then the positive feedback loop can amplify noise effectively and produce bursts of activity. In such a way the fate of the cell is determined in a stochastic manner, as shown in [14,15]. Such behavior splits the typical low transcription isogenic cell population into two groups. In the first group the Tat feedback loop produces new Tat particles (the “on” state). In the second group the Tat feedback loop is inactivated (the “off” state). This phenomenon was termed the phenotypic bifurcation (PheB). A series of carefully designed experiments were performed [14] to show that such behavior is indeed a product of intracellular noise, and is independent from extracellular perturbations. The possibility of a spontaneous latency decision caused by uncontrollable fluctuations of the Tat copy number has been clearly demonstrated.

### Current anti-latency treatments need to be improved

Even though several anti-latency drugs have been investigated, it is not clear how to administer them efficiently. For example, there is no consensus whether they should be administered aggressively (all at once), by repeated injection of smaller amounts, or constantly administered over a longer time period [4,5,21,22].

It is generally agreed upon that due to potential side effects of the treatment (e.g. toxicity), minimal dosages are preferable. A convincing argument for administering Minimal dosages is that too large amounts of the activation drug might release the virus beyond control, such that the usual highly active antiretroviral therapy (HAART) cannot contain it any longer [21]. Moreover, there are viral reservoirs, e.g., the brain, that are not easily accessible by HAART. In such organs it is very important to activate the virus gradually.

Clinical studies show that a global T-cell activation cannot eradicate the virus. Instead, it causes unwanted side effects [22]. An additional problem is that many of the activating agents are generic to a wide range of gene regulation processes; administering them is expected to show severe toxicity in the host. Thus the reduction of the dosage of anti-latency drugs appears desirable. In this context, noise-driven activation could be highly beneficial.

### Intrinsic fluctuations in protein copy numbers could be used to achieve more efficient activation

A therapy can be envisioned where several types of drugs work in synergy with the specific aim of moving the operating point of the virus into a noisy region, where the frequency of spontaneous virus activation would increase. Such therapy could be sustained for a longer time since, presumably, lower dosages of anti-latency drugs could be used. Targeting fluctuations in Tat copy number is very natural in this context, since the Tat protein is an essential part of the gene transcription machinery which produces new viral particles.

Fluctuations in *Tat* copy number have the potential to drive the activation. The up to date understanding of the feedback loop is that *Tat* controls transcriptional elongation rather than initiation. However, it seems that these two processes cannot be clearly separated, as it has been shown that an exogenous injection of *Tat* can activate the latent cell [14,23-27]. We note that a study on mice [25] showed no obvious side effects.

It is somewhat surprising that exogenous administration of Tat can in fact have a positive effect on the activation of infected cells. The lack of Tat molecules is just one of the many barriers to activation. If the transcription of the viral DNA is also blocked by other mechanisms that are not controlled by the *Tat* protein, the injection of exogenous Tat should not speed up the transcription process automatically. In fact, there are also cases where *Tat* by itself cannot reactivate the virus [28].

This supports a notion that in latently infected cells the transcription system is in a rather labile state, and that the factors affecting the transcription process do not work strictly in a binary on-off fashion. Such a system could be activated by spontaneous fluctuations in Tat copy number. Based on this insight, in the next section we will construct a mathematical machinery to identify useful strategies to achieve noise-assisted activation of the virus.

### A mathematical manual for the design of noise-based activation strategies

The central idea in the subsequent discussions is the concept of the “operating point” of the virus. The term will refer to a particular choice of the parameters that define the dynamics of the system, i.e., the virus and the cell it infects. In fact, one of the difficulties in combating HIV is that the integration of the virus into different sites gives rise to proviruses that have variable gene expression properties. It is conceivable that this results in different operating points of the virus. Therefore it is not immediately clear how a drug designed to achieve noise-based activation at a specific operation point would work consistently at other operating points. It is important to address this concern.

The manual we envision must contain a description of how (i) each anti-viral treatment affects an operating point, (ii) how the amounts of administered agents affect the magnitude of the off-set, and (iii) which anti-latency effect the induced off-set has. If the operating point of the virus is moved to regions where the latent state is less stable, the effect of antiviral drugs might be enhanced, which implies reduced dosage and shortened treatment. Below, each of these key considerations is mathematically formalized.

### Mathematical description of therapies and dosage

*Assumption I. The main function of each anti-latency agent is to alter the operating point of a virus:* Assume that the virus is at an operating point ${\vec{\lambda }}_{*}$, being a list of relevant parameters that describe the reaction system (the latent cell). If a certain amount of anti-latency agents is administered then this will off-set the operating point of the virus by $\vec{\Delta \lambda }$ and move it to a new operating point ${\vec{\lambda }}_{*}+\vec{\Delta \lambda }$. Thus mathematically, with every activation strategy one needs to associate the related displacement of the operating point it induces. It is possible that a treatment generates always the same displacement regardless of the operating point of the virus, but this does not necessarily need to be the case.

In this abstract sense a treatment of the HIV latency is a vector mapping $\vec{ℳ}$ from the space of operating points $\vec{\lambda }$ into the space of operating point off-sets

(1) $$\vec{\Delta \lambda }=\vec{ℳ}(\vec{\mathrm{\Delta Q}},\vec{\lambda })$$

where the vector $\vec{\mathrm{\Delta Q}}$ lists the amounts of anti-latency agents that are administered. This quantity can be viewed as an additional parameterization of the mapping. It is the same for every operating point. The condition

(2) $$\vec{ℳ}(\vec{\mathrm{\Delta Q}}=0,\vec{\lambda })=(0,0,0)$$

should be always satisfied, i.e., with nothing administered no operating point off-set is induced.

*Assumption II. The size of the operating point off-set depends on the dosage:* The size of the off-set $\vec{\Delta \lambda }$, to be denoted by $∥\vec{\Delta \lambda }∥$, is related to the amount of agents that are administered, to be denoted by $\left|\vec{\mathrm{\Delta Q}}\right|$. Clearly, these two quantities should be related. The simplest and rather general way to proceed is to assume that they are proportional to each other

(3) $$|\vec{\mathrm{\Delta Q}}|=\text{const}∥\vec{\Delta \lambda }∥$$

where the size of the off-set is defined as

(4) $$∥\vec{\Delta \lambda }∥=\sqrt{\Delta \lambda_{v}^{2}+\Delta \lambda_{u}^{2}+\Delta \lambda_{\epsilon }^{2}}$$

being the standard Cartesian norm of the vector $\vec{\Delta \lambda }$.

A possibility is to define $\left|\vec{\mathrm{\Delta Q}}\right|$ as the sum of its components. Another, more practically relevant, definition could involve some toxicity measure

(5) $$\left|\vec{\mathrm{\Delta Q}}|=T_{ℳ}(\vec{\mathrm{\Delta Q}}\right)$$

To avoid working with complicated metric spaces and projection procedures, the simplest possible relationship in the form of a proportionality law will be assumed.

### Probability of activation and related observables

The central quantity of interest is the probability of the activation of a latent cell in an observation interval *t*_0_<*t*^′^<*t*, to be denoted by *P*(*t*_0_,*t*).

*Assumption III. Off-sets should be induced to reduce the survival probability of a latent virus.* In strict mathematical terms *P*(*t*_0_,*t*) is a complex functional on the space of trajectories (histories). A history of the latent cell is defined as a set of individual trajectories for each particle type that define the biochemical content of the cell

(6) $$\left\{n_{1}(t^{\prime }),n_{2}(t^{\prime }),\cdots \;,n_{N}(t^{\prime })|t_{0}\leq t^{\prime }\leq t\right\}$$

where *n*_*i*_(*t*^′^) for *i*=1,2,⋯,*N* are copy numbers of relevant reactants (e.g. proteins) at time *t*^′^.

Intuitively, one expects that not all details of trajectories are important and that the activation probability depends strongly on a distinct feature of a history quantified by an observable (quantifier) *Φ*,

(7) $$P(t_{0},t)=\Pi (\Phi )$$

The observable *Φ* is a functional on the space of histories and *Π*(*Φ*) is a real valued function. Two examples of *Φ* will be discussed later. If the observable *Φ* is chosen well, the activation probability should depend on *Φ* in a threshold like manner,

(8) $$\Pi (\Phi )\approx \left\{\begin{array}{cc} 0 & \Phi <\Phi_{*}\\ 1 & \Phi \geq \Phi_{*} \end{array}\right)$$

where *Φ*_∗_ is a constant.

### The noise-free and noise-based activation concepts

**Figure 1:**
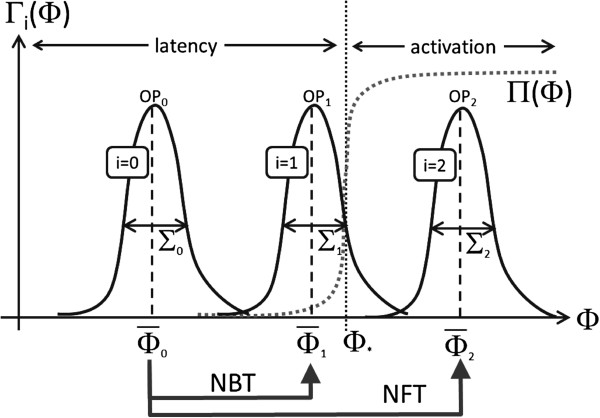
**The meaning of the noise-free and the noise-based activations.** The figure illustrates the meaning behind the noise-free and noise-based activations. All graphs were drawn by hand. *Γ*_*i*_(*Φ*) with *i*=0,1,2 are distribution functions for the observable *Φ* for three systems in three operating points: the latent cell *i*=0, the cell that has been activated using the noise-based therapy (*i*=1), and the cell that has been activated using noise-free therapy (*i*=2). ${\bar{\Phi }}_{i}$ and *Σ*_*i*_ are the means and the standard deviations. The noise free therapy (NFT) shifts the operating point of the virus from the stable (latent) operating point *OP*_0_ to the operating point *OP*2. This almost certainly activates the virus since ${\bar{\Phi }}_{2}-\kappa \Sigma_{2}\gg \Phi_{*}$. The noise-based therapy (NBT) induces the shift from the *OP*_0_ to the operating point *OP*_1_ where the activation is less certain but still possible since the tail of the *Γ*_1_ enters into the activation region and ${\bar{\Phi }}_{1}+\kappa \Sigma_{1}\approx \Phi_{*}$. Variable *κ* is a numerical factor close to 1.

Since trajectories are stochastic, the variable *Φ* is also stochastic and can be described by some probability distribution function *Γ*(*Φ*). Assume that two types of treatments have been designed which adjust the operating point of the stable latent virus (*OP*_0_) so that the respective distributions for *Φ* are obtained as depicted in Figure 1 (*OP*_1_, *OP*_2_). The two distinct scenarios will be referred to as the “noise-free” (NFA) and the “noise-driven (based)” (NBA) activations. The distributions are characterized by their respective means (${\bar{\Phi }}_{1}$, ${\bar{\Phi }}_{2}$) and standard deviations (*Σ*_1_, *Σ*_2_). The *NFA* activates with absolute certainty since ${\bar{\Phi }}_{2}\pm \Sigma_{2}\gg \Phi_{*}$. The NBA is less successful, since there are many instances where ${\bar{\Phi }}_{1}<\Phi_{*}$ but due to frequent fluctuations the threshold is reached often and ${\bar{\Phi }}_{1}+\Sigma_{1}\approx \Phi_{*}$. Thus in strict mathematical terms, a noise-free treatment ignores fluctuations and aims to establish an operating point such that $\bar{\Phi }\approx \Phi_{*}$. A noise-based treatment does not achieve the equality but there are frequent fluctuations that do, and $\bar{\Phi }+\Sigma \approx \Phi_{*}$

### Workings and effects of noise-free therapies

The purpose of a noise-free (NF) therapy ${\vec{ℳ}}_{\text{NF}}(\vec{\mathrm{\Delta Q}},{\vec{\lambda }}_{*})$ is to increase the quantify

(9) $$\Phi_{\text{NF}}({\vec{\lambda }}_{*})\equiv \bar{\Phi }({\vec{\lambda }}_{*})$$

as much as possible. Under the influence of the treatment with a small dosage the expected change of this quantity

(10) $$\Delta \Phi_{\text{NF}}(\vec{\Delta \lambda })\equiv \Phi_{\text{NF}}({\vec{\lambda }}_{*}+\vec{\Delta \lambda })-\Phi_{\text{NF}}({\vec{\lambda }}_{*})$$

can be approximated by

(11) $$\Delta \Phi_{\text{NF}}(\vec{\Delta \lambda })∼{\vec{\chi }}_{*}∙\vec{\Delta \lambda }$$

where

(12) $${\vec{\chi }}_{*}={\vec{\nabla }}_{\lambda }\Phi_{\text{NF}}(\vec{\lambda }){|}_{\vec{\lambda }={\vec{\lambda }}_{*}}$$

is the gradient computed at the operating point ${\vec{\lambda }}_{*}$.

Very likely, due to experimental constraints, not all off-sets can be realized. In an ideal situation where all off-sets can be induced, there is a class of treatments which are the most efficient. Such therapies should induce the off-set in the direction of the gradient

(13) $${\hat{\chi }}_{*}=\frac{{\vec{\chi }}_{*}}{∥{\vec{\chi }}_{*}∥}$$

as

(14) $$\vec{\Delta \lambda }={\vec{ℳ}}_{\text{NF}}(\vec{\mathrm{\Delta Q}},{\vec{\lambda }}_{*})\propto {\hat{\chi }}_{*}$$

Such a treatment will be referred to as optimal noise-free.

### Workings and effects of noise-based treatments

For a noise-based treatment ${\vec{ℳ}}_{\text{NB}}(\tilde{\mathrm{\Delta Q}},{\vec{\lambda }}_{*})$ the goal is to increase

(15) $$\Phi_{\text{NB}}({\vec{\lambda }}_{*})\equiv \bar{\Phi }({\vec{\lambda }}_{*})+\kappa \Sigma ({\vec{\lambda }}_{*})$$

where variable *κ* quantifies the typical size of a fluctuation. For a small dosage, the expected change of this quantity

(16) $$\Delta \Phi_{\text{NB}}(\vec{\Delta \lambda })\equiv \Phi_{\text{NB}}({\vec{\lambda }}_{*}+\vec{\Delta \lambda })-\Phi_{\text{NB}}({\vec{\lambda }}_{*})$$

can be approximated by

(17) $$\Delta \Phi_{\text{NB}}(\vec{\Delta \lambda })∼{\vec{\psi }}_{*}∙\vec{\Delta \lambda }$$

where

(18) $${\vec{\psi }}_{*}={\vec{\nabla }}_{\lambda }\Phi_{\text{NB}}(\vec{\lambda }){|}_{\vec{\lambda }={\vec{\lambda }}_{*}}$$

It is useful to partition this gradient further into a noise-free part and a noise-related part

(19) $${\vec{\psi }}_{*}={\vec{\chi }}_{*}+\kappa {\vec{\nu }}_{*}$$

where the noise related-part is given by

(20) $${\vec{\nu }}_{*}={\vec{\nabla }}_{\lambda }\Sigma (\vec{\lambda }){|}_{\vec{\lambda }={\vec{\lambda }}_{*}}$$

The precise value for *κ* depends on the character of the particle number distribution function and the confidence interval, but in here it will be taken as *κ*∼1 for simplicity reasons. Note that the effects of noise can be shut-off by taking *κ*=0.

Among all noise-based (NB) treatments a class of most efficient treatments exist, which induce the off-set in the direction

(21) $$\tilde{\Delta \lambda }={\vec{ℳ}}_{\text{NB}}(\tilde{\mathrm{\Delta Q}},{\vec{\lambda }}_{*})\propto {\hat{\psi }}_{*}$$

where

(22) $${\hat{\psi }}_{*}=\frac{{\vec{\psi }}_{*}}{∥{\vec{\psi }}_{*}∥}$$

Such a treatment will be referred to as optimal noise-based.

### The dose reduction coefficient can be used to quantify at which operating points the noise-based treatment is useful

Of particular interest is to find a proper combination of drugs that can achieve a maximal effect with a minimal dosage. We compare two arbitrary procedures ${\vec{ℳ}}_{\text{NF}}(\vec{\mathrm{\Delta Q}},{\vec{\lambda }}_{*})$ and ${\vec{ℳ}}_{\text{NB}}(\tilde{\mathrm{\Delta Q}},{\vec{\lambda }}_{*})$ with the requirement that

(23) $$\Delta \Phi_{\text{NF}}(\vec{\Delta \lambda })=\Delta \Phi_{\text{NB}}(\tilde{\Delta \lambda })$$

implying that both therapies induce the same change of the quantities they respectively try to maximize. The quantities are given in (9) and (15) respectively, and $\vec{\Delta \lambda }$ and $\tilde{\Delta \lambda }$ are the respective off-sets. For a given $\vec{\Delta \lambda }$ we wish to find the smallest possible vector $\tilde{\Delta \lambda }$ such that equation (23) holds:

(24) $$|\tilde{\mathrm{\Delta Q}}|=\text{min}\Leftrightarrow ∥\tilde{\Delta \lambda }∥=\text{min}$$

If the size of the vector $\tilde{\Delta \lambda }$ is smaller than the size of the vector $\vec{\Delta \lambda }$ then a noise based therapy with lower dosage is possible.

The minimization problem defined in (23) and (24) can be applied to decide whether a superior noise-based treatment exists, resulting in a lower dosage. The solution of the optimization problem is given by

(25) $$\tilde{\Delta \lambda }={\hat{\psi }}_{*}\;\frac{{\vec{\chi }}_{*}∙\vec{\Delta \lambda }}{∥{\vec{\psi }}_{*}∥}$$

where the dot denotes the scalar product. The ratio

(26) $$\Theta ({\vec{\lambda }}_{*},\vec{\Delta \lambda })\equiv \frac{∥\tilde{\Delta \lambda }∥}{∥\vec{\Delta \lambda }∥}=\frac{\left|{\vec{\chi }}_{*}∙\vec{\Delta \lambda }\right|}{∥{\vec{\psi }}_{*}∥∥\vec{\Delta \lambda }∥}$$

will be used to quantify the comparison between the two strategies. This quantity indicates the degree of the dose reduction for the treatment associated with a vector $\vec{\Delta \lambda }$. It does not depend on the size of the displacement vector $\vec{\Delta \lambda }$, only on its direction relative to the gradient of the mean.

In the case where the optimal noise-free treatment $\vec{\Delta \lambda }$ is aligned along the gradient of the mean, the amount of dose reduction becomes

(27) $$\Theta ({\vec{\lambda }}_{*})\equiv \frac{∥{\vec{\chi }}_{*}∥}{∥{\vec{\psi }}_{*}∥}=\frac{∥{\vec{\chi }}_{*}∥}{∥{\vec{\chi }}_{*}+\kappa {\vec{\nu }}_{*}∥}$$

to be referred to as the dose reduction coefficient. Ideally, $\Theta ({\vec{\lambda }}_{*})<1$ but the opposite is perfectly possible. When $\Theta ({\vec{\lambda }}_{*})>1$ noise-based therapy simply would not work as well as its noise-free counterpart.

### The space of operating points where a noise-based activation strategy can work

Now the mathematical manual will be used to find regions in the space of operating points of the latent virus where a noise-based activation is effective. To do this we focus on the *Tat* feedback loop. It is essential to model the noise of the *Tat* feedback loop, and to choose a relevant observable. The loop itself will be modelled in the simplest possible way as shown in the next subsection. The features of the system we wish to describe and the related observables are discussed subsequently.

### The resistor model of HIV latency

The “resistor model” of the HIV dormancy control has been suggested to explain how the lack of the *Tat* molecules maintains (stabilizes) the latent state [14,15]. This simplified description of the transcription machinery will be used for the theoretical analysis. The biochemical model [15] consists of entities (or particle types) *TatA* and *TatD* which denote the acetylated and deacetylated form of the *Tat* molecule respectively. To simply the notation *TatA* and *TatD* will be abbreviated as *A* and *D* respectively.

The model consists of the following chemical reactions. Each deacetylated *Tat* molecule can get acetylated with rate *α*,

(28) $$D\overset{\alpha }{\to }A$$

and each acetylated *Tat* molecules can get deacetylated with rate *β*,

(29) $$A\overset{\beta }{\to }D$$

Deacetylated *Tat* molecule decay with rate *δ*

(30) $$D\overset{\delta }{\to }\emptyset$$

An acetylated *Tat* molecule works as a transcription factor for the expression (production) of an additional *TatD* molecule,

(31) $$A\overset{k}{\to }A+D$$

It is clear that the model defined above neglects many biochemical details and is far from complete. For example, the mechanisms listed above are just a subset of all modifications of the Tat protein that occur in the cell [29]. Furthermore, a weak basal expression of *TatD* is continuously occurring [14] but this process will be neglected in the same way as in [15]. The model does not discriminate between mRNAs and proteins, either.

### A class of noise-based activation strategies

**Figure 2:**
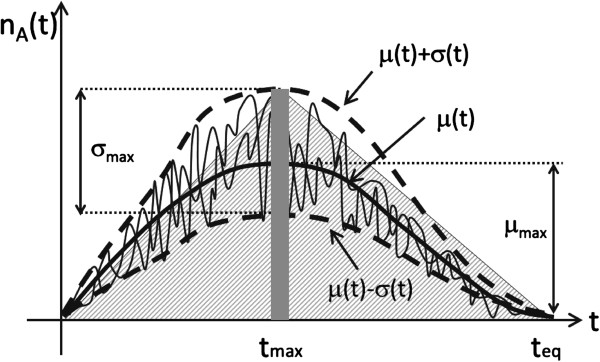
**The characteristics of a typical pulse.** The figure illustrates the key features of the activity pulse. All graphs were drawn by hand. The pulse lasts roughly *t*_eq_ and attains the maximum at roughly *t*≈*t*_max_. Additional key quantities of interest are the height of the pulse *μ*_max_, fluctuations around the peak described by the standard deviation *σ*_max_, and the surface under the curve. The patterned triangle can be used to approximate the shape.

The resistor model exhibits two states depending on the choice of the reaction rates. In the active state the feedback loop dominates the dynamics and all copy numbers increase. In the stable (dormant) state, bursts of activity last for a finite period of time during which the number of *TatA* molecules increases, reaches a maximum at *t*=*t*_max_, and then decays to zero. Such bursts of activity will be referred to as pulses. A pulse lasts typically for a time *t*_eq_. They rarely happen spontaneously and need to be initiated by injection of deacetylated *Tat*. This behavior is illustrated in Figure 2.

The stability condition has been formulated mathematically in [15]. The system is stable if

(32) αk<βδ

This condition is a fundamental property of the system. It defines the balance between the processes that produce and destroy *TatA* molecules. The system is stable if the production is slower than the destruction.

The key behavior of the resistor model we wish to exploit is related to pulses of activity. We investigate a hypothetical situation where *Tat* molecules are administered weakly at a constant rate in combination with an anti-viral drug and one or more operation point off-setting agents. The injection of *Tat* should trigger repeated pulses of activity. The anti-viral drug should reduce the copy number of viruses, while the off-setting agents move the operating point of the virus towards a nosier regime. The role of the off-setting agents is to boost fluctuations in the *Tat* expression levels so that the dosage of the anti-viral drug can be reduced.

### Two pulse characteristics as key observables

We now apply the mathematical machinery to compute the dose reduction coefficient for a wide range of reaction rate parameters (operating points) of the resistor model. It is necessary to identify an observable upon which the activation probability strongly depends. It will be assumed that the determining observable that governs the activation probability is the amount of viral *mRNAs* (proteins) produced by the feedback loop for the duration of a single pulse triggered by a single injection of *Tat*. If there are many viral particles that are ready for packaging and transport, the latent cell should activate.

The number of *tatD* mRNAs produced during the activation of the pulse can be computed by investigating how many molecules are produced by the *A*→*A*+*D* channel during the pulse duration. The expected amount of mRNAs produced in a small time interval *dt* is exactly given by *kn*_*A*_(*t*) *dt*. Integrating this quantity for the whole pulse duration gives

(33) $$\Phi_{\text{PS}}∼kA$$

where A

(34) $$A=\overset{\infty }{\underset{0}{\int }}\text{dt}n_{A}(t)$$

is the pulse surface (PS), i.e., the surface under the *n*_*A*_(*t*) curve (see Figure 2). This scenario will be referred to as *the pulse surface-dependent threshold scenario*, and when appropriate quantities computed in this context will be labelled by *PS* as in the example above.

There is no explicit experimental evidence that the surface is the one determining factor that most strongly influences activation. However, this idea is supported by several experimental studies. For example, in the experiment published in [15] it was argued that the duration of the activity pulse is likely to be important. The strength of the feedback loop regulates the duration of activity bursts. For less stable states the duration of such bursts increases dramatically, until they become equally long, and eventually longer than the life-time of the cell. Furthermore, in [30] it was experimentally shown that a stronger feedback implies more likely activation. Stronger feedback should correlate with the size of the surface A. Thus the first observable that will be used to implement the mathematical manual is the area A.

Describing the fluctuations of the area A is a very hard mathematical problem, since computing fluctuations involves the double integral over time of the particle density correlation function. This correlation function cannot be directly computed from lowest order moments of the particle number distribution function, only numerically at high computational cost. It is possible to simplify the mathematical treatment by focussing solely on the pulse height, which is much simpler to describe. Thus an additional observable will be considered, the maximum *Tat* copy number observed for a given trajectory. This scenario will be referred to as *the concentration-dependent threshold scenario*. The key quantity in this scenario is the pulse height (PH),

(35) $$\Phi_{\text{PH}}∼n_{A}(t_{\text{max}})$$

where *n*_*A*_(*t*) is the number of *A* at time *t* (see Figure 2). The quantities computed in this context will be labeled by *PH* as above.

While *Φ*_PH_ will be primarily used for illustrative purposes to gain qualitative understanding, the concentration-dependent threshold scenario does have practical significance. One expects that the number of *Tat* molecules in the cell correlates with the number of other viral proteins since their expression is encoded in the same gene. In that sense, high copy numbers of *Tat* protein should correlate with large activation probability. There are also some experimental indications in favour of the scenario.

Although there is no direct evidence that *Tat* on its own ensures activation, there is experimental evidence that exogenous injection of *Tat* can activate the latent cell [25]. Interestingly, there is also a report of the opposite [28]. Moreover, it has been suggested in several publications that *Tat* operates in a threshold dependent manner [6,8,31,32], but there is no direct experimental evidence for that. Interestingly, it is known that the *Rev* protein which controls export of viral mRNAs operates in a threshold dependent manner, and *Tat* drives the concentration of *Rev* above the threshold (See [15] for a discussion).

### Computation of the dose reduction coefficients for the concentration-dependent threshold scenario (the pulse height scenario)

The average number of acetylated *Tat* molecules at the peak *μ*_max_ and the related standard deviation of it *σ*_max_ are the key quantities that need to be computed. Figure 2 illustrates the meaning of these quantities. Both have to depend on the parameters that define an operating point of the latent cell,

(36) $$\begin{array}{ll} \mu_{\text{max}} & =\mu_{\text{max}}(\vec{\lambda })\; \end{array}$$

(37) $$\begin{array}{ll} \sigma_{\text{max}} & =\sigma_{\text{max}}(\vec{\lambda })\; \end{array}$$

The functions $\mu_{\text{max}}(\vec{\lambda })$ and $\sigma_{\text{max}}(\vec{\lambda })$ will be defined later on.

In this context the following choice for the observables of interest is the most natural:

(38) $$\Phi_{\text{NF}}(\vec{\lambda })=\mu_{\text{max}}(\vec{\lambda })$$

and

(39) $$\Phi_{\text{NB}}(\vec{\lambda })=\mu_{\text{max}}(\vec{\lambda })+\kappa \sigma_{\text{max}}(\vec{\lambda })$$

This results is the following dose reduction coefficient computed at an operating point of interest ${\vec{\lambda }}_{*}$:

(40) $$\Theta_{\text{PH}}({\vec{\lambda }}_{*})=\frac{∥{\vec{\mu }}_{*}^{\prime }∥}{∥{\vec{\mu }}_{*}^{\prime }+\kappa {\vec{\sigma }}_{*}^{\prime }∥}$$

where

(41) $${\vec{\mu }}_{*}^{\prime }\equiv {\vec{\nabla }}_{\lambda }\mu_{\text{max}}(\vec{\lambda }){|}_{\vec{\lambda }={\vec{\lambda }}_{*}}$$

and

(42) $${\vec{\sigma }}_{*}^{\prime }\equiv {\vec{\nabla }}_{\lambda }\sigma_{\text{max}}(\vec{\lambda }){|}_{\vec{\lambda }={\vec{\lambda }}_{*}}$$

Note that $\Theta ({\vec{\lambda }}_{*})$ depends on the gradients and not on the values:

(43) $$\begin{array}{ll} \mu_{*} & \equiv \mu_{\text{max}}({\vec{\lambda }}_{*})\; \end{array}$$

(44) $$\begin{array}{ll} \sigma_{*} & \equiv \sigma_{\text{max}}({\vec{\lambda }}_{*})\; \end{array}$$

### Computation of the dose reduction coefficients for the pulse-surface dependent threshold scenario

We try to estimate fluctuations of A from the knowledge of *μ*_max_ and *σ*_max_. The average surface can be estimated by approximating it with the area of the triangle emphasized in Figure 2. This area is roughly given by

(45) $$A(\vec{\lambda })\approx \frac{1}{2}\;t_{\text{eq}}(\vec{\lambda })\;\mu_{\text{max}}(\vec{\lambda })$$

where *t*_eq_ denotes the average equilibration time of the system. This quantity has to be a well-defined function of the operating point $t_{\text{eq}}=t_{\text{eq}}(\vec{\lambda })$.

The standard deviation of the area is much harder to estimate. A rough estimate for a typical fluctuation of this surface is given by

(46) $$\delta A(\vec{\lambda })\approx \frac{1}{2}\left(t_{\text{eq}}(\vec{\lambda })\delta n_{A}(t_{\text{max}})+\delta t_{\text{eq}}(\vec{\lambda })n_{A}(t_{\text{max}})\right)$$

where $\delta n_{A}(t_{\text{max}})∼\kappa \sigma_{\text{max}}(\vec{\lambda })$ is a typical fluctuation of the number of *A* at *t*=*t*_max_ and $\delta t_{\text{eq}}(\vec{\lambda })$ is the size of a typical fluctuation of the pulse duration. To avoid the mathematical complexity involved in computing $\delta t_{\text{eq}}(\vec{\lambda })$, it will be assumed that this quantity does not fluctuate.

These assumptions results in the following observable for the noise-free activation analysis,

(47) $$\Phi_{\text{NF}}(\vec{\lambda })\approx \frac{1}{2}\;\tau (\vec{\lambda })\;\mu_{\text{max}}(\vec{\lambda })$$

and the related noise-corrected quantity is given by

(48) $$\Phi_{\text{NB}}(\vec{\lambda })\approx \frac{1}{2}\tau (\vec{\lambda })\left[\mu_{\text{max}}(\vec{\lambda })+\kappa \sigma_{\text{max}}(\vec{\lambda })\right]$$

The re-scaled equilibration time

(49) $$\tau (\vec{\lambda })=k\;t_{\text{eq}}(\vec{\lambda })$$

measures the ratio between the length of the pulse duration and the time needed to produce a single *tat* mRNA. It equals roughly the number of viral particles produced during the single pulse. The definitions above result in the following dose reduction coefficient:

(50) $$\Theta_{\text{PS}}({\vec{\lambda }}_{*})=\frac{∥\tau_{*}\;{\vec{\mu }}_{*}^{\prime }+{\vec{\tau }}_{*}^{\prime }\;\mu_{*}∥}{∥\tau_{*}\;({\vec{\mu }}_{*}^{\prime }+\kappa {\vec{\sigma }}_{*}^{\prime })+{\vec{\tau }}_{*}^{\prime }\;(\mu_{*}+\kappa \sigma_{*})∥}$$

where

(51) $$\tau_{*}=\tau ({\vec{\lambda }}_{*}),{\vec{\tau }}_{*}^{\prime }=\vec{\nabla }\tau (\vec{\lambda }){|}_{\vec{\lambda }={\vec{\lambda }}_{*}}$$

## Methods

Thus, the required key quantities are the average number of *Tat* molecules at this peak, its standard deviation $\sigma_{\text{max}}(\vec{\lambda })$, and the typical time of the pulse duration scaled with the expression rate $\tau (\vec{\lambda })$.

### The mathematical description of noise: An overview of computing the mean and the standard deviation

The mean and the standard deviation can be computed from a few lowest order factorial moments of the particle number distribution function *P*(*n*_*A*_,*n*_*D*_,*t*) which specifies the probability that the system will be found in a state (*n*_*A*_,*n*_*D*_) at time *t*. The factorial moments are defined as

(52) ρx,y(t)=nAxx!nDyy!

where *x* and *y* are arbitrary positive integers, and the angular brackets denote the usual ensemble average of an arbitrary function (observable) *f*(*n*_*A*_,*n*_*D*_),

(53) $$\langle f(n_{A},n_{D})\rangle =\underset{n_{A},n_{D}}{\sum }f(n_{A},n_{D})P(n_{A},n_{D},t)$$

The mean and the variance are computed from the related factorial moments; *ρ*_*x*,*y*_(*t*) with *x*+*y*≤2:

(54) $$\begin{array}{ll} \mu_{A}(t) & =\rho_{1,0}(t)\; \end{array}$$

(55) $$\begin{array}{ll} \mu_{D}(t) & =\rho_{0,1}(t)\; \end{array}$$

(56) $$\begin{array}{ll} \sigma_{A}^{2}(t) & =\rho_{2,0}(t)+\rho_{1,0}(t)[1-\rho_{1,0}(t)]\; \end{array}$$

(57) $$\begin{array}{ll} \sigma_{D}^{2}(t) & =\rho_{0,2}(t)+\rho_{0,1}(t)[1-\rho_{0,1}(t)]\; \end{array}$$

Of particular interest will be the values of *μ*_*A*_(*t*) and *σ*_*A*_(*t*) at $t=t_{t_{\text{max}}}$. The equations of motion for the factorial moments needed to compute these quantities are discussed in the next section.

### The equation system for factorial moments

By using the procedure detailed in [33,34], it is possible to show that the equation system for the factorial moments is given by

(58) $$\begin{array}{c} {\dot{\rho }}_{x,y}(t)=\mathrm{\alpha x}\rho_{x-1,y+1}(t)\\ \;+(\beta +k)y\rho_{x+1,y-1}(t)\\ \;+\text{kxy}\rho_{x,y-1}(t)\\ \;-[(\alpha +\delta )y+\mathrm{\beta x}]\rho_{x,y}(t) \end{array}$$

In principle, such equation system forms an infinite hierarchy which for the present system decouples automatically. It is known that when all propensity functions in the stochastic formulation are linear with respect to the population count the computation of statistical moments is simple. For example, the equations for *ρ*_1,0_ and *ρ*_0,1_ form a closed system, and likewise the equations for *ρ*_2,0_, *ρ*_0,2_, and *ρ*_1,1_. It can be seen that the equations for *ρ*_*x*,*y*_ with *x*+*y*≤*ξ* form a closed system for every *ξ*=1,2,3,⋯. This analysis implies that the solution to a particular equation system is exact. Also, since the equation system for factorial moments up to order *ξ*=2 is exact, the values for the mean and the variance will be exact.

### Direct numerical integration should be avoided

In order to solve the equations it is necessary to integrate them from some initial condition from time *t=0* until *t*=*t*_max_. There are several reasons why a direct numerical integration should be avoided. First, the computational cost of a single time integration scales linearly with the inverse of a typical time step size used. A direct numerical integration is highly impractical, since to construct the phase diagrams, the noise measures have to be computed at many points in the reaction rate space.

Second, there will be a need to compute derivatives of the mean and the standard deviation with respect to the reaction rates. If obtained numerically, his would multiply the computational effort by a factor two or more, depending on which technique is used to numerically calculate the derivatives.

We have found a procedure to avoid the numerical integration and yet obtain an analytic approximation of these quantities. The details of how *μ*_max_≡*μ*_*A*_(*t*_max_) and *σ*_max_≡*σ*_*A*_(*t*_max_) are computed are given in the next section. Both quantities depend on ratios of the reaction rates. Accordingly, it is useful to re-scale all rates to obtain dimensionless parameters, e.g., by rate *α*. This reduces the four dimensional Cartesian space of reaction rates defined by tuples (*α*,*β*,*δ*,*k*) into a three-dimensional Cartesian space defined by tuples $\left(\bar{\beta }\equiv \beta /\alpha ,\bar{\delta }\equiv \delta /\alpha ,\bar{k}\equiv k/\alpha \right)$. Thus,

(59) $$\begin{array}{ll} \mu_{\text{max}} & =f_{\mu }(\bar{\beta },\bar{\delta },\bar{k})\; \end{array}$$

(60) $$\begin{array}{ll} \sigma_{\text{max}} & =f_{\sigma }(\bar{\beta },\bar{\delta },\bar{k})\; \end{array}$$

where the functions *f*_*μ*_ and *f*_*σ*_ are detailed in the next section. When convenient the subscript “max” will be omitted. To simplify notation we will use only *μ* and *σ* instead of *μ*_max_ and *σ*_max_.

### Details of computing the mean and the standard deviation

In this section it will be shown how to obtain the equations of motion for factorial moments and the functional forms for the mean and the standard deviation.

### A procedure for avoiding numerical integration

The numerical analysis of the equations of motion (not shown) suggests that the following parameterization is useful:

(61) $$\begin{array}{ll} \rho_{1,0}(t) & \equiv a(t)\; \end{array}$$

(62) $$\begin{array}{ll} \rho_{0,1}(t) & \equiv d(t)\; \end{array}$$

(63) $$\begin{array}{ll} \rho_{2,0}(t) & \equiv a{\left(t\right)}^{2}+a(t)(\varphi_{A}(t)-1)\; \end{array}$$

(64) $$\begin{array}{ll} \rho_{0,2}(t) & \equiv d{\left(t\right)}^{2}+d(t)(\varphi_{D}(t)-1)\; \end{array}$$

(65) $$\begin{array}{ll} \rho_{1,1}(t) & \equiv \sqrt{a(t)d(t)}\varphi_{\text{AD}}(t)\; \end{array}$$

where *φ*_*A*_ and *φ*_*D*_ are the noise strengths for *A* and *D* particle types since it is trivial to see that

(66) $$\begin{array}{ll} \sigma_{A}^{2}(t) & =a(t)\varphi_{A}(t)\; \end{array}$$

(67) $$\begin{array}{ll} \sigma_{D}^{2}(t) & =d(t)\varphi_{D}(t)\; \end{array}$$

The variable *φ*_*AD*_(*t*) is a generalization of the noise strength concept for a pair of particle types.

A numerical integration of the equations of motion shows that for large times *η*=*σ*/*μ*→*∞* but, in the same limit, the noise strengths become constant,

(68) $$\begin{array}{ll} \underset{t\to \infty }{\lim}\varphi_{A}(t) & =\varphi_{A}^{*}\; \end{array}$$

(69) $$\begin{array}{ll} \underset{t\to \infty }{\lim}\varphi_{D}(t) & =\varphi_{D}^{*}\; \end{array}$$

(70) $$\begin{array}{ll} \underset{t\to \infty }{\lim}\varphi_{\text{AD}}(t) & =\varphi_{\text{AD}}^{*}\; \end{array}$$

This can be used to obtain approximations for the noise strengths at *t*=*t*_max_.

First, it is possible to construct an algebraic system of equations for these quantities by studying the asymptotic limit of the ODE system for *φ*_*A*_(*t*), *φ*_*D*_(*t*) and *φ*_*AD*_(*t*). This implies that finding the asymptotic values for the noise strengths can be reduced to an algebraic problem. Second, for the Poisson initial condition the noise strengths are given by

(71) $$\begin{array}{ll} \underset{t\to 0}{\lim}\varphi_{A}(t) & =1\; \end{array}$$

(72) $$\begin{array}{ll} \underset{t\to 0}{\lim}\varphi_{D}(t) & =1\; \end{array}$$

This can be seen from the fact that for the Poisson initial condition $\sigma_{A}^{2}(t=0)=a(t=0)$ and $\sigma_{D}^{2}(t)=d(t=0)$ which after using the noise strength definitions (66) and (67) automatically leads to the above result. Accordingly, given that the particle number distribution function is Poisson-like at *t*≈0, one can expect that *φ*_*A*_(*t*) and *φ*_*D*_(*t*) vary within a finite interval.

A single cell can be infected by more than one virus particle. The number of viral particles that infect a given cell (the multiplicity of infection) varies randomly and is usually Poisson-distributed. Accordingly, in what follows it will be assumed that the initial particle number distribution function is Poisson-like.

With the assumptions at hand, the values of *φ* are known at two time instances, around *t=0* and *t*≈*t*_eq_. Here and in the following *t*_eq_ denotes the time after which the *φ* variables reach their asymptotic values. This information can be used to obtain a very crude approximation for the noise strengths in the form of a linear interpolation between the points *t=0* and *t*=*t*_eq_. Once the interpolation has been carried out, the values of the noise strengths at *t*=*t*_max_ are then given by

(73) $$\begin{array}{ll} \varphi_{A}(t_{\text{max}}) & =1+(\varphi_{A}^{*}-1)\frac{t_{\text{max}}}{t_{\text{eq}}}\; \end{array}$$

(74) $$\begin{array}{ll} \varphi_{D}(t_{\text{max}}) & =1+(\varphi_{D}^{*}-1)\frac{t_{\text{max}}}{t_{\text{eq}}}\; \end{array}$$

This is the approximation that will be used to compute noise strengths at the time instance where the number of the acetylated *Tat* particles reaches maximum.

There is no *a priori* reason why the noise strengths should vary linearly with time. We have inspected several curves where noise strengths were computed numerically to see whether the time dependence is linear. Interestingly, while the time dependence is not strictly linear it seems that the approximation used is qualitatively correct. We performed more rigorous tests of such an approximation by comparing it with the results of a numerical integration for wide range of parameters and found reasonable agreement.

The time *t*_eq_ can be found (not shown) by computing the eigenvalues of the matrix that defines the ODE system for the first and the second order moments. The smallest eigenvalue governs the relaxation time which is given by

(75) $$t_{\text{eq}}=\frac{2}{q_{1}-q_{2}}$$

where

(76) $$q_{1}=\alpha +\beta +\delta$$

and

(77) $$q_{2}=\sqrt{q_{1}^{2}-4(\beta \delta -\mathrm{\alpha k})}$$

### The equation system for noise strengths

By using the parameterization just introduced it is possible to obtain the equations of motion for the means and the noise strengths. In addition, to make the analytic analysis easier it is useful to map the means onto the Poincaré sphere:

(78) $$\begin{array}{ll} a(t) & =\frac{1}{z(t)}\; \end{array}$$

(79) $$\begin{array}{ll} d(t) & =\frac{u(t)}{z(t)}\; \end{array}$$

This is a useful mathematical technique to perform asymptotic analysis (see [35] and references therein). By using the new variables the equations for the means

(80) $$\begin{array}{ll} \dot{a}(t) & =\mathrm{\alpha d}(t)-\mathrm{\beta a}(t)\; \end{array}$$

(81) $$\begin{array}{ll} \dot{d}(t) & =(\beta +k)a(t)-(\alpha +\delta )d(t)\; \end{array}$$

become

(82) $$\begin{array}{ll} Ż(t) & =[\beta -\mathrm{\alpha u}(t)]z(t)\; \end{array}$$

(83) $$\begin{array}{lll} \dot{u}(t) & =\beta +k+[\beta -(\alpha +\delta )]u(t)\; & \;\\ & \;\;\;\;\;-\mathrm{\alpha u}{\left(t\right)}^{2}\; \end{array}$$

By combining (58) with (61-65) it is possible to obtain the equations for the noise strengths that are given by

(84) $$\begin{array}{lll} {\dot{\varphi }}_{A}(t)=\; & 2\alpha \sqrt{u(t)}\varphi_{\text{AD}}(t)\; & \;\\ & -[\beta +\mathrm{\alpha u}(t)](\varphi_{A}(t)-1)-2\mathrm{\alpha d}(t)\; \end{array}$$

(85) $$\begin{array}{lll} {\dot{\varphi }}_{D}(t)=\; & 2\frac{\beta +k}{\sqrt{u(t)}}\varphi_{\text{AD}}(t)-\left[\alpha +\delta +\frac{\beta +k}{u(t)}\right]\; & \;\\ & \times [\varphi_{D}(t)-1]-2(\beta +k)a(t)\; \end{array}$$

(86) $$\begin{array}{lll} {\dot{\varphi }}_{\text{AD}}(t)=\; & \alpha \sqrt{u(t)}[\varphi_{D}(t)-1]\; & \;\\ & +\frac{\beta +k}{\sqrt{u(t)}}[\varphi_{A}(t)-1]+\frac{k}{\sqrt{u(t)}}\; & \;\\ & -\left[\alpha +\beta +\delta +\mathrm{\alpha u}(t)+\frac{\beta +k}{u(t)}\right]\frac{\varphi_{\text{AD}}(t)}{2}\; & \;\\ & +\mathrm{\alpha d}(t)\sqrt{u(t)}+\frac{\beta +k}{\sqrt{u(t)}}a(t)\; \end{array}$$

It will be shown later that the terms proportional to *a(t)* and *d(t)* also drop out in the asymptotic limit when the stability condition is satisfied.

### Locating the peak region

The value for *t*_max_ can be easily found by requiring that $\dot{a}(t_{\text{max}})=0$. The equation system (80-81) has to be solved with the initial condition *a*(0)=0 and *d*(0)=*d*_0_>0 which results in the expression for *a*(*t*) (not shown), the derivative of which is required to vanish. This gives

(87) $$t_{\text{max}}=\frac{1}{q_{2}}\ln\frac{q_{1}+q_{2}}{q_{1}-q_{2}}$$

One can also find that

(88) $$\mu_{\text{max}}=a(t_{\text{max}})=d_{0}\frac{2\alpha }{q_{1}+q_{2}}{\left(\frac{q_{1}+q_{2}}{q_{1}-q_{2}}\right)}^{\frac{2q_{2}}{q_{1}-q_{2}}}$$

In order to see what happens when relatively few deacetylated *Tat* molecules are injected the equation above will be used with *d*_0_=1.

### Computing the asymptotic noise strengths

The equation (83) does not involve the variable *z(t)* and can be used to obtain the asymptotic value for the ratio *d(t)/a(t)* as time approaches infinity. This differential equation has one stable fixed point in the physical region *u(t)>0*. The fixed point value for *u(t)*, *i.e.*, lim*t*→*∞u*(*t*)=*u*^∗^, is given by

(89) $$u^{*}=\frac{\sqrt{{\left(\alpha -\beta +\delta \right)}^{2}+4\alpha (\beta +k)}-(\alpha -\beta +\delta )}{2\alpha }$$

Note that there are no restrictions on the reaction rates, only that they are positive real numbers. This implies that in this model the ratio of the number of acetylated and deacetylated *Tat* molecules approaches a constant value regardless on whether the system is stable or not.

The asymptotic (fixed point) value of *z(t)* can be determined by considering how the term *β*−*αu*(*t*) on the right hand side of Eq. (82) behaves as time becomes large. From (89) one can see that

(90) $$\underset{t\to \infty }{\lim}[\beta -\mathrm{\alpha u}(t)]=\frac{\beta \delta -\mathrm{\alpha k}}{\Lambda }$$

where *Λ* is a strictly positive constant that depends on the values of the reaction rates. Its exact numerical value is not relevant for the discussion and the formula for *Λ* will not be shown. For large times one has

(91) $$z(t)\propto \exp\left[\frac{\beta \delta -\mathrm{\alpha k}}{\Lambda }t\right]$$

When *βδ*−*αk*>0, *z(t)* grows exponentially fast, implying that the means approach zero. This condition is fully equivalent to the stability condition (32). On the other hand, when *βδ*−*αk*0, *z(t)* approaches zero exponentially fast, which implies that the average copy numbers become infinite. In both cases the ratio of the means becomes constant and is given by *u*^∗^.

For a stable system one can neglect the terms proportional to *a(t)* or *d(t)* in Eqs. (84-86) when *t*→*∞*. This results in a linear system of equations for the asymptotic values of the noise strengths given by

(92) $$\begin{array}{ll} 2\alpha \sqrt{u^{*}}\varphi_{\text{AD}}^{*} & =(\beta +\alpha u^{*}){\bar{\varphi }}_{A}^{*}\; \end{array}$$

(93) $$\begin{array}{ll} \frac{\beta +k}{\sqrt{u^{*}}}\varphi_{\text{AD}}^{*} & =\left(\alpha +\delta +\frac{\beta +k}{u^{*}}\right)\frac{{\bar{\varphi }}_{D}^{*}}{2}\; \end{array}$$

$$\begin{array}{lll} & \;\left[\alpha +\beta +\delta +\alpha u^{*}+\frac{\beta +k}{u^{*}}\right]\frac{\varphi_{\text{AD}}^{*}}{2}\; & \; \end{array}$$

(94) $$\begin{array}{ll} & \;=\alpha \sqrt{u^{*}}{\bar{\varphi }}_{D}^{*}+\frac{\beta +k}{\sqrt{u^{*}}}{\bar{\varphi }}_{A}^{*}+\frac{k}{\sqrt{u^{*}}}\; \end{array}$$

where

(95) $$\begin{array}{ll} {\bar{\varphi }}_{A}^{*} & =\varphi_{A}^{*}-1\; \end{array}$$

(96) $$\begin{array}{ll} {\bar{\varphi }}_{D}^{*} & =\varphi_{D}^{*}-1\; \end{array}$$

Finally, *σ* can be computed as

(97) $$\sigma_{\text{max}}=\sigma (t_{\text{max}})=\sqrt{a(t_{\text{max}})\varphi_{A}^{*}}$$

where *a*(*t*_max_) is given in (88) and $\varphi_{A}^{*}$ is obtained by solving (93-94). All algebraic equations have been solved by using the Mathematica package (not shown).

## Results

### A hyperbolic coordinate system facilitates understanding

The space of the reaction rates is multi-dimensional and relatively hard to visualize. Numerical tests showed that it is advantageous to re-parameterise the space of $\left(\bar{\beta },\bar{\delta },\bar{k}\right)$ tuples by using the hyperbolic-like coordinate system,

(98) $$\begin{array}{ll} \frac{\beta }{\alpha } & =ve^{u}\; \end{array}$$

(99) $$\begin{array}{ll} \frac{\delta }{\alpha } & =ve^{-u}\; \end{array}$$

(100) $$\begin{array}{ll} \frac{k}{\alpha } & =v^{2}(1-\epsilon )\; \end{array}$$

Accordingly, an operating point will be the triple

(101) $$\vec{\lambda }=(v,u,\epsilon )$$

In fact, this set of coordinates is very intuitive which can be appreciated by analysing the meaning of the inverse transformation.

The coordinate *v* is given by

(102) $$v=\sqrt{\beta \delta }/\alpha$$

This quantity measures to which extent the reactions which remove acetylated *Tat* dominate over the reaction that produces it. The coordinate

(103) $$u=\frac{1}{2}\ln\frac{\beta }{\delta }$$

measures the relative contribution of the reactions that remove acetylated *Tat* molecules. These two coordinates naturally form a hyperbolic coordinate system. The variable

(104) $$\epsilon =1-\frac{\mathrm{k\alpha}}{\beta \delta }$$

is particularly important for several reasons. First, it measures how intensive the transcription process (the production of acetylated *Tat* molecules) is. This coordinate also measures how far in the ility region the operating point of the virus is placed. For example, *ϵ*=0 at the border of the stab stability region when *αk*=*βδ*. Furthermore, *ϵ*≈1 when the stability condition is strongly satisfied, *i.e.* when *βδ*≫*kα*. Thus, in the stable region *ϵ* attains values in the interval between zero and one.

This variable could potentially be used to experimentally quantify the degree of the latency for a given cell. For example, the literature suggests that for a latent virus its operating point has to lie in the region of stability where *ϵ*>0. Otherwise, for *ϵ*0, an infected cell would lyse relatively fast. Thus, it seems necessary that a treatment meant to activate a latent virus has to move its operating point into the unstable region where *ϵ*0 or at least sufficiently close to the instability boundary *ϵ*=0.

An example of a typical operating point can be obtained from the resistor model. From the experimental values [15] for the reaction rates *α*_*R*_=0.5/day, *β*_*R*_=5/day, *δ*_*R*_=2/day, and *k*_*R*_=5/day one can compute the corresponding operating point

(105) $${\vec{\lambda }}_{R}=(6.325,0.458,0.750)$$

The symbol “*R*” emphasizes the resistor model operating point.

### Regions of parameter space where noise is large

**Figure 3:**
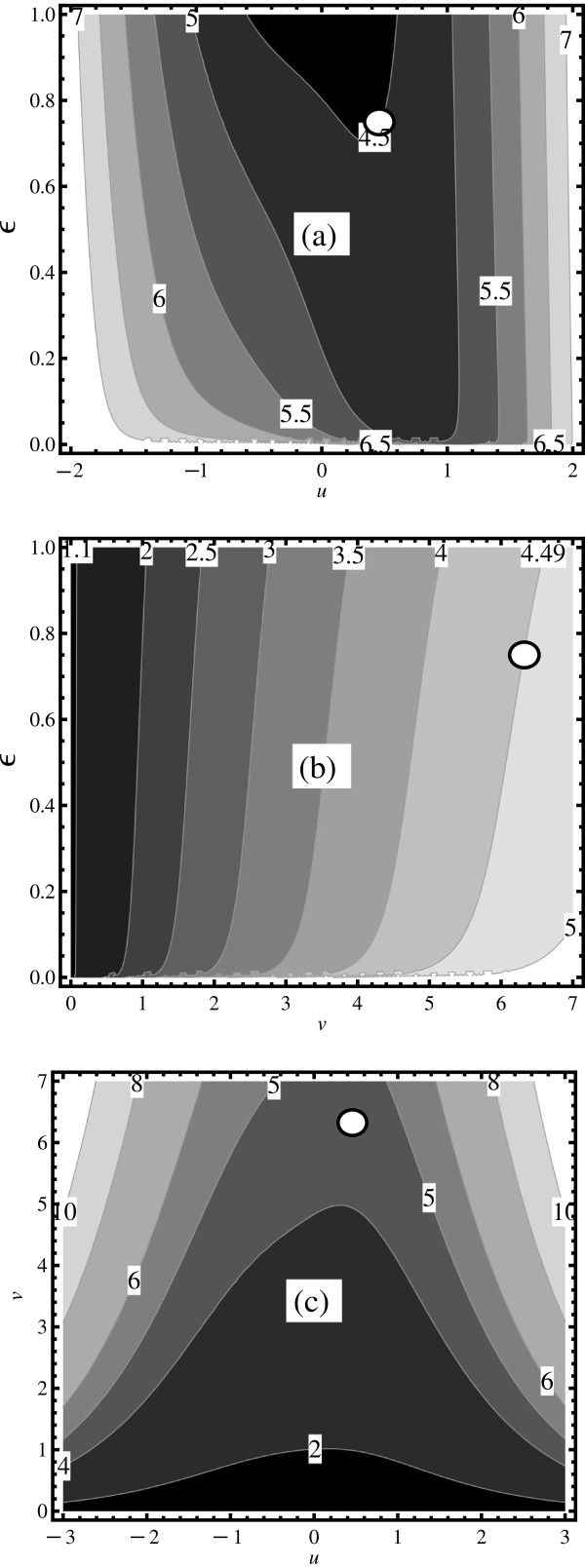
**Contour plots of *σ */ *μ * in three hyperplanes: (a) for ( *v *=*v***_***R***_**, *u *, *ϵ *), (b) for ( *v *, *u *=*u***_***R***_**, *ϵ *), and (c) for ( *v *, *u *, *ϵ *=*ϵ***_***R***_**).** The position of the resistor model operating point is marked by the white circle. Contour lines are labelled by their respective *σ*/*μ* values (square boxes). Lighter (darker) regions indicate where noise does (does not) dominate the dynamics.

Perhaps the biggest advantage with the hyperbolic coordinate system suggested is that the *v* dependence is very easy to visualize. Figures 3a, 3b, and 3c depict how the coefficient of variation *η*=*σ*/*μ* depends on the reaction rate parameters. Figures 3a and 3b demonstrate that when *ϵ* approaches the border of stability (*ϵ*→0) the coefficient of variation approaches infinity. Figures 3b and 3c show that if *v* is increased, the coefficient of variation always increases, no matter which values for *u* and *ϵ* are chosen. Figures 3a and 3c indicate that if noise is to be exploited for a treatment, one should design drugs that could move the operating point of the virus away from the regions around *u*≈0. These plots show in which regions of the parameter space the effects of noise are expected to dominate. Superficial analysis of these figures would suggest that one should choose rates *β* and *δ* as different as possible from each other, since this should increase the amount of noise relative to the mean. However, in doing so one might move the operating point such that despite the increase in fluctuations the threshold cannot be reached. A more quantitative analysis is needed in order to identify useful noise-based strategies. This is illustrated by a case study, where we suggest how the mathematical manual developed above can be used to guide experimental design.

### A case study: A strategy for improving noise-free histone deacetylase inhibitor treatment by moving the operating point of the resistor system to a noisier region

A typical anti-latency strategy in the HIV therapy context is to increase the transcription rate *k*, for example in treatments based on the use of histone deacetylase inhibitors [36]. These molecules open up the chromatin environment such that the transcription factors needed for viral expression can attach easier to their respective binging sites. Several molecules have been suggested as drug candidates. Valproic acid, trichostatin A, vorinostat (SAHA), and many more have been reviewed in [36], and references therein. Some of these molecules are rather toxic. In the following we investigate how toxicity could be reduced by lowering their dosage at a typical operation point, e.g. ${\vec{\lambda }}_{R}$. In order to analyse the effects of various treatments it is useful to explicitly compute the gradients of the mean and the standard deviation at this operating point. We will focus on the conceptually simpler concentration-dependent threshold scenario.

The expressions for the derivatives are not shown. Their numerical values are given by:

(106) $$\begin{array}{ll} {\vec{\mu }}_{R}^{\prime } & =-(0.00808,0.0161,0.00911)\; \end{array}$$

(107) $$\begin{array}{ll} {\vec{\sigma }}_{R}^{\prime } & =-(0.0182,0.0582,0.0597)\; \end{array}$$

By administering a certain amount of an anti-latent drug, e.g., SAHA, which only shifts *k*_*R*_ to *k*_*R*_+*Δk* and leaves *α*_*R*_, *β*_*R*_, and *δ*_*R*_ unchanged, the operating point would be shifted by

(108) $${\vec{\Delta \lambda }}_{\text{SAHA}}=(0,0,1)\Delta \epsilon$$

where *Δϵ* is arbitrary but controlled by the amount of SAHA administered. From Eq. (26) one can see that a more effective noise-based therapy with SAHA as the primary drug exists, since $\Theta_{\text{PH}}({\vec{\lambda }}_{R},{\vec{\Delta \lambda }}_{\text{SAHA}})=0.087$, which is smaller than one. From (25) one obtains the exact value of the off-set that has to be achieved in order to produce the same gain in the number of *Tat* molecules through noise-based therapy

(109) $${\tilde{\Delta \lambda }}_{\text{SAHA}}=(0.0219,0.0618,0.0573)\Delta \epsilon$$

Different changes in the reaction rates have to be used in order to generate these two vastly different offsets. For example, let us investigate which changes in the transcription rate *k* are needed to achieve the changes in (108) and (109).

From (98-100) follows that

(110) $$\begin{array}{ll} \frac{\Delta \bar{\beta }}{\bar{\beta }} & =\frac{\mathrm{\Delta v}}{v}+\mathrm{\Delta u}\; \end{array}$$

(111) $$\begin{array}{ll} \frac{\Delta \bar{\delta }}{\bar{\delta }} & =\frac{\mathrm{\Delta v}}{v}-\mathrm{\Delta u}\; \end{array}$$

(112) $$\begin{array}{ll} \frac{\Delta \bar{k}}{\bar{k}} & =2\frac{\mathrm{\Delta v}}{v}-\frac{\Delta \epsilon }{1-\epsilon }\; \end{array}$$

where $\bar{\beta }=\beta /\alpha$, $\bar{\delta }=\delta /\alpha$, and $\bar{k}=k/\alpha$. For the noise-free treatment based on SAHA we assume that

(113) $$\begin{array}{ll} \Delta \alpha & =0\; \end{array}$$

(114) $$\begin{array}{ll} \Delta \beta & =0\; \end{array}$$

(115) $$\begin{array}{ll} \Delta \delta & =0\; \end{array}$$

(116) $$\begin{array}{ll} \mathrm{\Delta k} & >0\; \end{array}$$

In such a case the equations (110-112) and (113-116) imply that both *Δv* and *Δu* are zero, and the following relationship between *Δk* and *Δϵ* holds

(117) $$\mathrm{\Delta k}=-20\Delta \epsilon \;{\text{day}}^{-1}$$

However, in the case of the noise-based therapy we do not know a priori the values for $\tilde{\Delta \alpha },\;\tilde{\Delta \beta },\;\tilde{\Delta \delta }$, and $\tilde{\mathrm{\Delta k}}$. However, we do know that $\tilde{\mathrm{\Delta v}}=0.0219\Delta \epsilon ,\;\tilde{\mathrm{\Delta u}}=0.0618\Delta \epsilon$, and $\tilde{\Delta \epsilon }=0.0573\Delta \epsilon$. Again, for simplicity reasons we assume that $\tilde{\Delta \alpha }=0$ which upon using (110-112) gives the off-sets that need to be used in the noise-based treatment

(118) $$\begin{array}{ll} \tilde{\Delta \beta } & =0.326\Delta \epsilon \;{\text{day}}^{-1}\; \end{array}$$

(119) $$\begin{array}{ll} \tilde{\Delta \delta } & =-0.117\Delta \epsilon \;{\text{day}}^{-1}\; \end{array}$$

(120) $$\begin{array}{ll} \tilde{\mathrm{\Delta k}} & =-1.11\Delta \epsilon \;{\text{day}}^{-1}\; \end{array}$$

By comparing (117) and (120) one sees that the dose of the primary drug can be reduced roughly twenty times (assuming the linear relationship between the dose change and the transcription rate change). The price one has to pay is that additional drugs have to be administered which reduce *β* and increase *δ* (note that *Δϵ* is negative). It is not unrealistic that this can be eventually verified experimentally, e.g. in the context of the kinetics experiments on LTR [14,15].

Even if the optimal noise-free treatment would be applied in this case, noise-based treatment would still result in dose reduction. An immediate use of Eq. (27) shows that $\Theta_{\text{PH}}({\vec{\lambda }}_{R})=0.192$.

Note that $\Theta_{\text{PH}}({\vec{\lambda }}_{R},{\vec{\Delta \lambda }}_{\text{SAHA}})<\Theta_{\text{PH}}({\vec{\lambda }}_{R})$ since the SAHA therapy is not optimal. As the optimal noise free (gradient based) therapy is more efficient than the SAHA treatment, the corresponding noise based therapy would yield a smaller dose reduction.

This case study shows that it is sufficient to inspect the value of the dosage reduction coefficient $\Theta_{\text{PH}}(\vec{\lambda })$ in order to test whether a noise-based treatment would be beneficial at an operating point $\vec{\lambda }$.

### Regions of parameter space where noise-driven activation is possible

**Figure 4:**
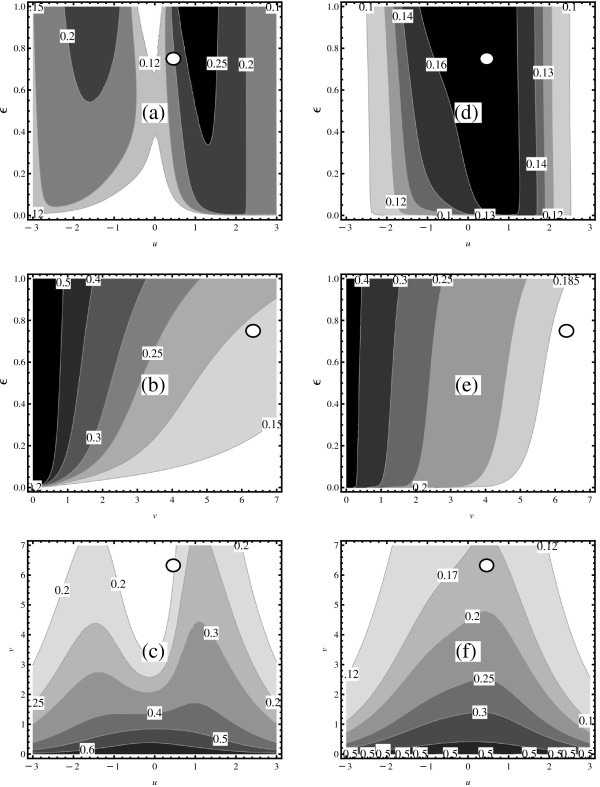
**Phase diagrams for the dose reduction coefficient.** The panels **(a-c)** and **(e-f)** depict contour plots of the dose reduction coefficients $\Theta_{\text{PH}}(\vec{\lambda })$ and $\Theta_{\text{PS}}(\vec{\lambda })$ respectively. For both quantities three hyperplanes are considered: **(a)** and **(d)** for (*v*=*v*_*R*_,*u*,*ϵ*), **(b)** and **(e)** for (*v*,*u*=*u*_*R*_,*ϵ*), **(c)** and **(f)** for (*v*,*u*,*ϵ*=*ϵ*_*R*_). The position of the resistor model operating point is marked by the white circle. In all plots for lighter (darker) regions the dose reduction coefficient has relatively small (large) values. In lighter (darker) regions the noise-based treatment is (not) advantageous over noise free treatment. For operating points that are located in light regions the noise-based treatment can bring relatively large dosage reductions while in the dark regions it might not be that advantageous if compared to a noise-free treatment.

To identify regions in parameter space where noise-based activation is beneficial, we determine how $\Theta (\vec{\lambda })$ depends on the operating point for the two scenarios of interest. The panels (a, d), (b, e), and (c, f) in Figure 4 depict the contours of *Θ*(*v*_*R*_,*u*,*ϵ*), *Θ*(*v*,*u*_*R*_,*ϵ*), and *Θ*(*v*,*u*,*ϵ*_*R*_) for (*Θ*=*Θ*_PH_, *Θ*=*Θ*_PS_) respectively.

A striking result of this visual analysis is that regions where $\Theta (\vec{\lambda })>1$ could not be found within the hyperplanes. For both scenarios, we computed the dose reduction coefficient for 10000 randomly selected operating points, and found always *Θ*1. Moreover, for the PH scenario, at all points the angle between ${\vec{\mu }}_{*}^{\prime }$ and ${\vec{\sigma }}_{*}^{\prime }$ is always in the ±*π*/2 interval, i.e., the vectors point in roughly the same direction. *It appears that the noise-based activation is advantageous everywhere, regardless which value for κ* is chosen. The degree of the dose reduction is clearly controlled by *κ*. Note that the related graphs for (*Θ*_PH_), (*Θ*_PS_) are roughly the same, which implies that the conclusion is generic. There are some differences that are worth discussing.

The contours in panels (a) and (d) differ in the middle region, and likewise for panels (c) and (f). There are even some regions where a relatively large dose reduction is possible. Figure 4a indicates that an efficient dosage reduction can be achieved in the regions around *u*≈0 where *ϵ* is either very large or very small. This does not hold for panel (d). However, both (a) and (d) panels suggest that the noise based therapy is advantageous in the region where the absolute value of *u* is very large.

Figures 4b and 4e show that for fixed *u* the largest dose reduction can be reached for very small values of *ϵ* and very large values of *v*. Figures 4c and 4f indicate that the noise-based activation can be useful in the regions where *v* is large and where either *u*→*∞* or *u*→−*∞*. The panels do not agree in the middle region.

### The linear theory yields qualitative predictions when operating point off-sets are not small

**Figure 5:**
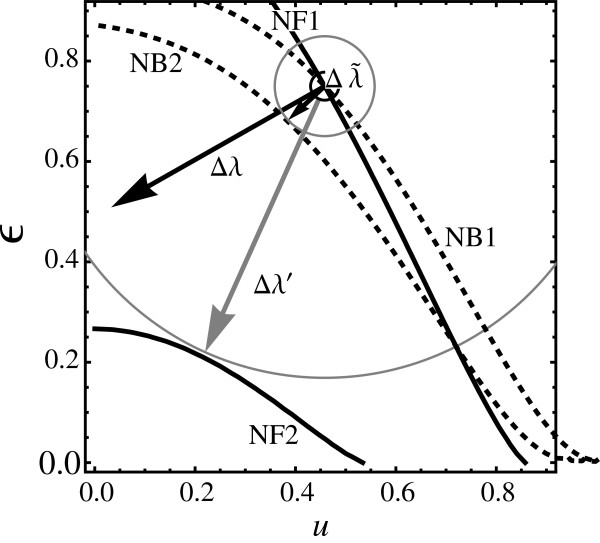
**Reaction rate dependence of*μ***_**max**_** and*μ***_**max**_**+*σ***_**max**_** in a ( *u *, *ϵ *) hyperplane.** Contour plots of *μ*_max_ and *σ*_max_+*μ*_max_ in the (*u*,*ϵ*) plane with *v*=*v*_*R*_. The full lines labelled NF1 and NF2 are two contours of *μ*_max_, the two dashed lines labelled NB1 and NB2 are contours of *σ*_max_+*μ*_max_. The operating point of the resistor model is represented by the white circle. The contour values for the NF1 and NB1 contours are chosen so that these lines pass through the operating point of the resistor model. The contours labelled NF2 and NB2 have been off-set from NF1 and NB1 by the same *ΔN*. The black arrows labelled $\vec{\Delta \lambda }$ are the projections of the operating point offsets which need to be achieved in order to induce change in *μ*_max_ and *μ*_max_+*σ*_max_ respectively by *ΔN*. For comparison, the grey arrow labelled ${\vec{\Delta \lambda }}^{\prime }$ is the shortest possible path from the operation point to NF2.

We provide a non-infinitesimal analysis for a particular operating point, and investigate how it differs from the corresponding linear analysis. To do this we will use the technically simpler PH scenario. This will be illustrated by studying how both *μ*_max_ and *μ*_max_+*σ*_max_ depend on *u* and *ϵ* for fixed *v* (Figure 5). One particular hyperplane has been chosen, where the size of the *u*- and *ϵ*- components of the off-set in Eq. (109) is somewhat larger than the *v*-component.

Several contour lines are shown: (NF1) *μ*_max_=*μ*_1_, (NF2) *μ*_max_=*μ*_1_+*ΔN*, (NB1) *μ*_max_+*σ*_max_=*μ*_1_+*σ*_1_, and (NB2) *μ*_max_+*σ*_max_=*μ*_1_+*σ*_1_+*ΔN* where *μ*_1_=0.05378, *μ*_1_+*σ*_1_=0.064536, and *ΔN*=0.010756.

The long black arrow denotes the linearized noise-free off-set that needs to be induced in order to shift the mean from *μ*_max_ to *μ*_max_+*ΔN*. The long grey arrow is the shortest non-infinitesimal noise-free off-set that needs to be induced to move from the operating point to the NF2 contour line. The large grey circle (only in part visible) starts touching NF2 exactly at the point where the arrow meets the contour line. The short black arrow denotes the noise-based off-set that needs to be induced in order to shift *μ*+*σ* by *ΔN*. The small grey circle denotes the minimal circle that touches NB2.

The length ratio between the black vectors is specified exactly by the dose reduction coefficient $\Theta ({\vec{\lambda }}_{R})=0.192$. The grey arrow (the exact off-set) does not match its black counterpart neither in length nor direction. Clearly, the size of the noise-free offset $\vec{\Delta \lambda }$ needed to achieve the shift in *μ* is too large for the linearized version of the problem. This approximation is more satisfactory for the noise-based treatment since the related noise-based off-set $\tilde{\Delta \lambda }$ is still relatively small. The linearized theory provides qualitative results for non-infinitesimal off-sets and that can be used in practise to guide future experiments.

### A few comments on the robustness of the results with regard to the model extension

The model used in here cannot capture all phenomena that might be important. The first feature of the model that can be clearly improved is its complexity. For example, we made no distinction between mRNAs and proteins. It has been found that after integration into the genome the HIV promoter makes mRNAs in random bursts of transcriptional activity [37,38]. Moreover, each mRNA makes proteins in translational bursts. This suggests that there might be other sources of noise in the system that were not considered in this work.

Also, a rather phenomenological model was used for the probability of activation and the related observable. There is clearly a need for refinement. It is possible that the probability of the virus activation depends on other features of the system, e.g., the amount of relative fluctuations. This is rather speculative, based upon indications in the literature that *Tat* operates also outside the Tat feedback loop. *Tat* is a multi-functional protein that is involved in other intracellular processes [32,39,40] and there is certainly the possibility that it influences cell homeostasis in a more complex manner than discussed here. For example, in addition to acetylation, *Tat* undergoes several other post-translational modifications and interacts with several other proteins. It is currently not entirely clear how this might affect gene expression noise on one hand, and the activation probability on the other. Post-translational modifications or other interactions could buffer, or propagate noise in levels of *Tat* into noise in gene expression. The question remains whether such effects could be beneficial in bringing the operating point closer to the stability boundary. Out of all parameters in the model, such effects will very likely exert strong influence on the decay constant *δ* of the deacetylated *Tat* and cause it to fluctuate in time. One could speculate that in such a situation both *u* in *v* will start fluctuating, though *u,* being essentially the natural logarithm of the ratio between *β* and *δ*, will fluctuate much less. This implies that *v* fluctuates towards smaller values, away from the noise-rich regions, while *u* and *ϵ* are kept essentially constant (e.g. see the phase diagrams). Such changes in *v* could reduce the activation probability, and need to be further investigated.

We now briefly discuss possible effects of chromatin on the operating point of a virus. The transcription rate *k* will likely be influenced the most. This implies that instead of analysing the effect of a drug on a single operating point one should investigated a set of operating points. Such points should be distributed around the region where *v*≈*v*_*R*_, *u*≈*u*_*R*_ and where *ϵ*∈[0,1]. Accordingly, a noise-based treatment designed to move the operating point into a noisier region will work uniformly on all points in this set. This would always result in a relatively large dose reduction; Figure 4, panels (a), (b), (d) and (e).

## Conclusions

We have designed a generic mathematical manual of how to approach the problem of the HIV latency in a quantitative manner, accompanied by an example of how to use this manual. We formalised several concepts that are vaguely defined or understood only intuitively. The first key concept is the notion of an “operating point” of the virus and how it is affected by the action of a drug. The second key concepts of the mathematical formalism is the notion of a particular observable that strongly affects the activation probability. We suggested a mathematical way of describing how each therapy affects an operating point and how this in turn influences the observable that controls the activation probability. The third key ingredient is a dose reduction coefficient, which can be used to quantitatively compare various therapies.

We have suggested and investigated two rather general strategies for the virus activation, the noise-free and the noise-based strategy. In the first approach, anti-latency agents are administered such that the activation happens with almost absolute certainty. In order to achieve such certainty, possibly unreasonable quantities of drugs need to be administered. In the second approach, drugs are administered in such a way that the activation is less certain but still happens with a relatively large probability. The idea behind the noise-based strategy is to reduce the quantity of drugs that need to be administered in order to achieve activation.

The mathematical manual is rather generic. To demonstrate how the mathematical manual can be used, we have focused on the simplest possible model of the *Tat* protein feedback loop, the most important part of the the HIV latency control. Based on the structure of the loop we suggested a class of noise-based activation strategies. We envision such an activation strategy in a procedure where one constantly supplies exogenous *Tat* at a very small rate, and adds a combination of anti-latency drugs that would off-set the operating point of the virus towards noisier regions.

In this context we considered two observables and could compute the dose reduction coefficient for both cases to answer the fundamental question: for which operating points the noise-base therapy is advantageous over the noise-free therapy in the sense of possible dosage reduction? This addresses the practical problem of reducing effects of toxicity during the anti-latent treatment. Three phase diagrams were constructed to explain what controls the noise, and how this control can be used to battle latency. Our analysis of the phase diagrams indicates that the noise-based therapy is always advantageous, no matter which operating point the virus adopts. This results holds regardless of which observable is targeted. This is the major result of our analysis.

The mathematical manual is currently based on rather qualitative assumptions, but it is very generic and can be easily extended and refined. For example, we have used the assumption of small operating point offsets in order to linearize the theory. One can easily consider non-infinitesimal off-sets, as demonstrated in the example. We discussed several possible extensions which are left for future work.

In summary, we suggest an activation principle where intrinsic noise is considered a *feature benefiting treatment*. We showed that such strategy should be efficient for any latent cell.

## Competing interests

Both authors declare that they have no competing interests.

## Authors’ contributions

ZK has defined the research theme, developed the model, and performed computations. ZK and AJ analysed the results, discussed, and prepared the manuscript. Both authors read and approved the final manuscript.
